# Prenatal exposure to endocrine disrupting chemicals in relation to thyroid hormone levels in infants – a Dutch prospective cohort study

**DOI:** 10.1186/1476-069X-13-106

**Published:** 2014-12-10

**Authors:** Marijke de Cock, Michiel R de Boer, Marja Lamoree, Juliette Legler, Margot van de Bor

**Affiliations:** Faculty of Earth and Life Sciences, VU University, Section Health and Life Sciences, De Boelelaan 1085, 1081 HV Amsterdam, The Netherlands; Faculty of Earth and Life Sciences, Institute for Environmental Studies, VU University, De Boelelaan 1085, 1081 HV Amsterdam, The Netherlands

**Keywords:** Endocrine disruptors, Fetal basis of adult disease, Thyroxine, Prenatal exposure

## Abstract

**Background:**

Endocrine disrupting chemicals (EDCs) present in the environment may disrupt thyroid hormones, which in early life are essential for brain development. Observational studies regarding this topic are still limited, however as the presence of chemicals in the environment is ubiquitous, further research is warranted. The objective of the current study was to assess the association between exposure markers of various EDCs and thyroxine (T4) levels in newborns in a mother-child cohort in the Netherlands.

**Methods:**

Exposure to dichlorodiphenyldichloroethylene (DDE), three di-2-ethylhexyl phthalate (DEHP) metabolites, hexachlorobenzene (HCB), polychlorinated biphenyl (PCB)-153, perfluorooctanesulfonic acid (PFOS), and perfluorooctanoic acid (PFOA) was determined in cord plasma or breast milk, and information on T4 levels in heel prick blood spots was obtained through the neonatal screening programme in the Netherlands. Linear regression models were composed to determine associations between each of the compounds and T4, which were stratified for gender and adjusted for a priori defined covariates.

**Results:**

Mean T4 level was 86.9 nmol/L (n = 83). Girls in the highest quartile of DDE and PFOA exposure showed an increased T4 level compared to the lowest quartile with both crude and fully adjusted models (DDE > 107.50 ng/L, +24.8 nmol/L, 95% CI 0.79, 48.75; PFOA > 1200 ng/L, +38.6 nmol/L, 95% CI 13.34, 63.83). In boys a lower T4 level was seen in the second quartile of exposure for both PFOS and PFOA, however after fully adjusting the models these associations were attenuated. No effects were observed for the other compounds.

**Conclusion:**

DDE and perfluorinated alkyl acids may be associated with T4 in a sex-specific manner. These results should however be interpreted with caution, due to the relatively small study population. More research is warranted, as studies on the role of environmental contaminants in this area are still limited.

**Electronic supplementary material:**

The online version of this article (doi:10.1186/1476-069X-13-106) contains supplementary material, which is available to authorized users.

## Background

Thyroid hormones (TH) play a significant role during development, both during the fetal period as well as after birth. They are essential for neurodevelopment as they regulate genes involved in neuronal cell differentiation and myelination [1]. Abnormal TH homeostasis early in life therefore may have long-term consequences. Congenital hypothyroidism is the most common cause of mental retardation and is associated with amongst others high birth weight, deafness, and spasticity [2, 3]. Even small changes in maternal thyroid hormones may affect brain development, as maternal thyroid stimulating hormone (TSH) is inversely associated with offspring IQ [4]. Also other outcomes related to thyroid function, such as birth weight, may be affected by changes in maternal thyroid hormone levels within the normal range [5].

Hormones, including thyroid hormones, are sensitive to external factors such as chemicals which are present in the environment. Exposure to endocrine disrupting chemicals (EDCs) such as polybrominated diphenyl ethers (PBDEs), polychlorinated biphenyls (PCBs) and perfluorinated alkyl acids (PFAAs) have been associated with effects on TH homeostasis. Prenatal exposure to PBDE mixture DE-71 in rats has been associated with reduced T4 levels in both males and females [6, 7]. Male rats prenatally exposed to PBDE and polychlorinated biphenyls (PCBs), either as a single compound or as a mixture, also showed reduced T4 levels [8, 9]. Similar results were observed in prenatally exposed female rats [10]. Also gestational perfluorooctanesulfonic acid (PFOS) exposure was associated with reduced T4 levels in rats [11, 12]. Human studies on prenatal environmental exposures and thyroid hormones, and T4 in particular, are still scarce. Prenatal exposure to BDE-100 measured in cord blood of Taiwanese newborns was associated with an increase in thyroxine/3,5,3′-triiodothyronine (T4/T3) ratio [13], and also for BDE-154 measured in breast milk a positive correlation with cord blood log T4 was observed [14]. However, for BDE-99 a decrease in free T4 (fT4) levels was reported in the same study. Furthermore Kim et al. [15] observed that cord blood PFOS and perfluorooctanoic acid (PFOA) exposure were negatively associated with cord blood T4 levels [15]. However after adjusting the models for covariates, most relationships lost significance. Also Darnerud et al. who looked at early life exposure to PCBs and DDE in breast milk observed no associations with thyroid hormones in a population of Swedish newborns [16].

Results from previous studies are not consistent, and as neonatal TH levels may depend on various factors such as birth weight, gestational age, and mode of delivery, it is important to consider the timing of sampling [17]. Neonatal screening programs use heel prick blood spots sampled several days after birth, which is advantageous compared to e.g. cord blood as TH levels may be less affected by stress of the delivery [18]. The objective of this study was therefore to assess the association between exposure markers of various chemicals determined in cord blood and T4 levels determined as part of the national neonatal screening programme. For this purpose data from a newly established mother-child cohort in the Netherlands was used: the LINC study (Linking EDCs in maternal nutrition to child health). The compounds included were dichlorodiphenyldichloroethylene (DDE), polychlorinated biphenyl (PCB)-153, and hexachlorobenzene (HCB), of which health effects have been studied frequently, as well as some relatively ‘new’ compounds: metabolites of di(2-ethylhexyl) phthalate (DEHP), including mono(2-ethyl-5-carboxypentyl)phthalate (MECPP), mono(2-ethyl-5-hydroxyhexyl)phthalate (MEHHP), and mono(2-ethyl-5-oxohexyl)phthalate (MEOHP), as well as PFOS, and PFOA.

## Materials and methods

### Study procedures and subjects

Participants from the LINC study were recruited between January 2011 and January 2013, through six midwifery clinics in the area of Zwolle in the Netherlands. The community Zwolle is located in the ‘Salland’ area, which is characterized by agriculture and stock-breeding and a relatively low level of urbanization. As only one hospital is available to serve this community, child births were relatively easy to track. Women were invited to participate during the first antenatal visit to the midwife and were considered eligible for participation if they were able to fill out Dutch questionnaires. Twin pregnancies and major congenital anomalies were reasons for exclusion, however no participant was excluded because of these criteria. Signed informed consent was obtained from every participant. Cord blood and breast milk were collected for determination of markers of exposure to several endocrine disrupting chemicals. The national neonatal screening programme was contacted for data on T4 measured in heel prick blood samples. Information on birth weight, gestational age, and parental anthropometry was obtained from the midwives, and questionnaires were administered during pregnancy to collect information on parental health and lifestyle, and previous pregnancies. The study was approved by the medical ethics committee of the VU University medical centre in Amsterdam.

### Thyroxine

The National Institute for Public Health and the Environment was approached in order to obtain data on T4 as measured in the neonatal screening programme. Permission from the parents was ascertained for this specific part of the study. Blood samples were collected on filter paper through heel puncture which was performed on average between day 4 and day 7 after birth. Total T4 was determined by means of Autodelfia [19].

### Chemical exposure

These methods have been previously published [20]. Umbilical cord blood was collected immediately after birth when the health of mother and child was ascertained. Midwives and nurses were instructed to collect as much blood as possible in EDTA tubes through manual expression. The blood was delivered to the lab within twelve hours by a courier in case of home delivery or by someone from the hospital staff in case of delivery at the hospital. At the lab, cord blood was centrifuged for 10 minutes at 2000 g. after which the plasma layer was transferred to plasma tubes. Plasma was stored at -80°C.

Breast milk was collected in the second month after birth (mean [SD] weeks after birth: 6.3 [2.5]). In total a minimum of 100 mL. was collected, spread over five to ten days to minimize the burden to the mothers in case of low milk flow. Mothers were instructed to note the dates on which they collected a sample and to store the milk in the freezer in between sampling days. They were allowed to use a breast pump for collection.

Compounds were analysed in cord plasma. For DDE and HCB we also analysed breast milk samples from mothers for whom an insufficient amount of cord blood was available. PFOA and PFOS were analysed by applying isotope dilution and large volume injection using an on-line trapping column coupled to liquid chromatography and triple quadropole mass spectrometry. The breast milk samples were extracted with solid phase extraction using Oasis WAX cartridges. For the cord plasma samples, the proteins were precipitated by adding methanol and centrifuging the mixture prior to injection onto the analytical system.

After drying both the cord plasma and the breast milk samples with Kieselguhr, the organochlorine pesticides p,p’-DDE, HCB and PCB153 were extracted with a mixture of dichloromethane and hexane. Cleanup of the extracts was done using sulphuric acid silica columns and for the analysis gas chromatography with mass spectrometric detection in negative chemical ionization mode was used.

Di-2-ethylhexyl phthalate (DEHP) exposure was analysed by analysis of three secondary metabolites mono(2-ethyl-5-carboxypentyl) phthalate (MECPP), mono(2-ethyl-5-hydroxyhexyl) phthalate (MEHHP), and mono(2-ethyl-5-oxohexyl) phthalate (MEOHP). Enzymatic deconjugation was carried out, and after addition of the internal standards used for isotope dilution, the breast milk samples were extracted using Oasis MAX cartridges, while for the cord plasma samples a simple protein precipitation step using formic acid was applied. The extracts were analysed after large volume injection using an on-line trapping column coupled to liquid chromatography and triple quadropole mass spectrometry. Problems due to the occurrence of contamination of the breast milk samples are to be expected for mono(2-ethylhexyl)phthalate (MEHP), the hydrolytic monoester of DEHP, that has shown to also be formed in the matrix due to the residual activity of enzymes (lipases, esterases), even after prolonged storage periods at -20°C. Therefore, MEHP is a very unreliable parameter for the assessment of DEHP exposure and should not be used for breast milk. In contrast, MECPP, MEHHP, and MEOHP are not susceptible to contamination as they are major metabolites of DEHP created by the liver. They are only formed in vivo and serve as a reliable parameter for DEHP exposure.

The coefficient of variation for the chemicals measured, was 16 – 17%. More information on chemical analysis, including limits of quantification and quality control parameters, is given in Additional file 1, p. 2–4.

### Covariates

Covariates were selected based on literature [5, 21–25]. Each participant was asked for thyroid gland related health problems (yes or no) and use of related medication during pregnancy (yes or no), by means of questionnaire.

Birth weight was measured after birth by a midwife or a nurse and data were subsequently obtained from registries of the midwives. Newborns were put on the weighing scale without a diaper and birth weight was determined when the infant was in a calm state. Weighing scales were provided by the midwives and were calibrated daily.

Weight and length of the mother and father were measured by the midwife at inclusion, approximately 10–12 weeks in pregnancy. Measurement of maternal weight was repeated at 36 weeks in pregnancy to determine gestational weight gain. Midwives received strict instructions on how to perform these measurements as well as a measuring tape which was attached to a wall in the midwife’s office. Weighing scales were provided by the midwife and were calibrated daily. Gestational age was determined by midwives by means of ultrasound. Mode of delivery (caesarean section, yes or no) was recorded by the midwife.

Questionnaires were administered to collect information on birth date of the mother, parity, maternal smoking during the first trimester (yes or no), and alcohol intake during the first trimester (drinks per week).

### Data-analysis

Data analysis was performed using SPSS version 20 [26]. Separate linear regression models were composed for each compound. To take into account gender-specific effects of exposure, models were stratified for gender by means of interaction terms which were added to the models. For each compound exposure values below the limit of quantification (LOQ) were replaced by LOQ/√2 [27]. As DDE accumulates in lipid, the following conversion factor was used to transform levels in breast milk to levels in cord blood [27].


The conversion factor for DDE was based on what has been published for other European cohorts. For PCB-153 measured in cord plasma, over 50% of the samples had an exposure level that was below LOQ. As PCB-153 was determined predominantly in cord blood it was decided to include PCB-153 as a dichotomous variable (>LOQ vs. <LOQ). None of the compounds showed a linear association with T4 levels, therefore each exposure was split up in quartiles and included as dummy variables in the model. Models were initially adjusted for health problems related to the thyroid gland, use of thyroid medication, birth weight, and caesarean section. Fully adjusted models also included gestational weight gain, gestational age, parity, smoking, alcohol, maternal BMI, and maternal age at birth. Each of these covariates was checked for linearity with T4 and was included in the models regardless of the degree of confounding. As gestational weight gain is mostly seen as a confounder for lipophilic compounds, and as birth weight is considered to be associated with maternal thyroid hormones, we performed sensitivity analyses for these variables by excluding them from the models. The distribution of T4 was checked for normality and outliers by means of histograms. For each model the residuals were plotted and checked as well for normal distribution. Effect modification by smoking was also taken into consideration and was included if the interaction term was significant (p < 0.05).

## Results

In total 148 women were included, of whom 14 subjects dropped-out. Due to failed sample collection or insufficient sample volumes, exposure data were not available for an additional 51 children, resulting in the inclusion of 83 mother-child pairs for analysis. A description of the LINC study population is presented in Table 1. Mean T4 level was 85.6 nmol/L in boys and 89.6 nmol/L in girls, and mean birth weight was 3632 and 3546 grams in boys and girls respectively. Caesarean section was performed in 2.4% of births, and maternal thyroid disease as well as use of related medication was reported by one participant.

**Table 1:** **Population characteristics (n = 83)**

	Boys (n = 52)		Girls (n = 31)	
	Mean ± SD *	Min - max	Mean ± SD*	Min - max
T4 (nmol/L)	85.6 ± 20.0	42 - 138	89.6 ± 14.9	68 – 117
Birth weight (g.)	3632.1 ± 501.5	2130 – 4950	3546.3 ± 397.6	2710 – 4350
Gestational age (weeks)	39.7 ± 1.5	34.1 – 41.6	40.1 ± 1.0	36.6 – 42.0
Small for gestational age (n)	3 (5.8%)		1 (3.3)	
Large for gestational age (n)	5 (9.6)		2 (6.7)	
Parity (nulliparous, n)	20 (40.0%)	0 – 4	10 (32.3%)	0 – 3
Caesarean (yes, n)	2 (3.8%)	-	0	-
Thyroid disease (yes, n)	0	-	1 (3.3)	-
Thryoid medication (yes, n)	0	-	1 (3.3)	-
BMI mother (start pregnancy, kg/m^2^)	23.7 ± 3.4	17.8 – 32.5	23.0 ± 4.3	18.8 – 36.5
Gestational weight gain (kg.)	12.7 ± 4.2	1.0 – 23.0	12.1 ± 5.3	-6.0 – 20.0
Age mother (years)	33.0 ± 4.9	23.0 – 40.0	32.0 ± 3.4	23 – 36
Smoking (first trimester, yes, n)	2 (3.8%)	-	2 (6.5%)	-
Alcohol (yes, n)	2 (3.8%)	-	2 (6.5%)	-

^*^Values are mean ± SD unless stated otherwise.

An overview of exposure levels of various compounds in cord plasma as well as breast milk is given in Table 2. For PCB-153, DDE, and HCB, wet weight levels in breast milk were significantly higher than in cord plasma. However, lipid adjusted levels were similar, reflecting the high fat content of the breast milk. For these compounds the percentage of samples with exposure levels above the LOQ was also higher in breast milk than in cord blood. Concentrations of HCB in cord blood were < LOQ in more than 98% of the samples. Therefore HCB was not included in the analysis.

**Table 2:** **Exposure levels in cord plasma and/or breast milk**

Compound		N	Mean	Median	Range	LOQ	<LOQ (%)
**PCB-153**							
Cord plasma	- ng/L	51	35.40	28.28	22.63 – 96.00	21 – 43	57.7
	- ng/g lipid	51	36.02	30.00	17.95 – 88.89	14 – 53	57.7
**DDE**							
Cord plasma	- ng/L	51	114.48	79.00	37.48 – 470.00	33 – 73	23.1
	- ng/g lipid	51	115.98	81.97	28.83 – 580.25	23 – 86	23.1
Breast milk	- ng/L	24	2379.58	1895.00	400.00 – 11390.00	9.20 – 13.00	0
	- ng/g lipid	24	59.20	44.10	12.11 – 277.80	0.13 – 0.53	0
Total^a^	- ng/L	75	100.70	74.50	14.53 – 470.00		
**HCB**							
Cord plasma	- ng/L	51	44.33	44.55	28.28 – 78.00	40 – 79	98.1
	- ng/g lipid	51	46.23	45.85	27.27 – 82.11	25 – 96	98.1
Breast milk	- ng/L	24	622.92	655.00	300.00 – 1060.00	9.20 – 13.00	0
	- ng/g lipid	24	15.94	14.94	10.65 – 25.85	0.16 – 0.68	0
**MECPP**							
Cord plasma	- ng/mL	64	0.31	0.27	0.11 – 1.00	0.13 – 0.28	7.8
**MEHHP**							
Cord plasma	- ng/mL	64	0.33	0.27	0.10 – 1.00	0.14 – 0.27	9.4
**MEOHP**							
Cord plasma	- ng/mL	64	0.29	0.23	0.12 – 0.87	0.17 – 0.33	25.0
**PFOA**							
Cord plasma	- ng/L	64	943.44	885	200 – 2700	50 – 140	0
**PFOS**							
Cord plasma	- ng/L	64	1616.88	1600	570 – 3200	44 – 140	0

^a^For total DDE, cord plasma exposure data were merged with breast milk exposure levels converted to cord plasma levels.

All three DEHP metabolites as well as the PFAAs had relatively high quantification rates. In contrast to what was observed for PCB-153, DDE, and HCB, levels of PFOS and PFOA were higher in cord plasma compared to breast milk.

Associations with T4 were observed for total DDE, PFOS, and PFOA, depending on the sex of the infant (Tables 3 and 4). Girls in the highest quartile of total DDE exposure showed significantly increased T4 levels compared to the lowest exposed group for crude as well as adjusted analyses, while for boys no difference in T4 levels were detected across quartiles. Boys in the second and third quartile of PFOS exposure had significantly lower T4 levels than the reference group with the crude model (respectively -22.1 nmol/L, 95% CI: -38.75 to -5.48; and -16.8 nmol/L, 95% CI -33.47 to -0.21). After partially adjusting the model, only the second quartile remained significantly lower than the reference group, but in the fully adjusted model also this association disappeared. In girls no effect of PFOS exposure on T4 levels was observed, however for PFOA the highest quartile of exposure showed increased T4 levels with the crude as well as the adjusted models. Mean T4 levels were 22.8 nmol/L (95% CI 0.86 to 44.79) higher compared to the reference group when no adjustments for covariates were made. This difference increased to 38.6 nmol/L (95% CI: 13.34 to 63.83) in the fully adjusted model. In boys no effect of PFOA exposure on T4 was observed, except for the second quartile of exposure, which had a significantly lower T4 level than the reference group (-20.3 nmol/L, 95% CI: -37.29 to -3.24). However, the adjusted analyses showed attenuated and even opposite associations that were no longer statistically significant. No significant associations with T4 were observed for the DEHP metabolites and for PCB-153 (Table 5). Removing birth weight or gestational weight gain from the models did not affect the results (not shown).

**Table 3:** **Regression coefficients for various exposures in boys (in quartiles) and T4 (nmol/L)**

	N	Q1	Q2		Q3		Q4	
Compound			β (95% CI)	p-value	β (95% CI)	p-value	β (95% CI)	p-value
**DDE (Total, ng/L)**		**<41.80**	**41.80 – 74.50**		**74.51 – 107.50**		**>107.50**	
Crude	70	Ref	-2.1 (-17.33, 13.23)	0.789	-8.5 (-23.51, 6.43)	0.258	2.1 (-12.27, 16.42)	0.773
Model A^1^	65	Ref	-3.3 (-20.00, 13.33)	0.690	-6.6 (-22.60, 9.31)	0.407	2.8 (-12.05, 17.58)	0.710
Model B^2^	61	Ref	10.5 (-13.94, 34.90)	0.389	2.9 (-17.00, 22.71)	0.771	8.0 (-10.68, 26.77)	0.389
**MECPP (ng/mL)**		**<0.22**	**0.22 – 0.27**		**0.28 – 0.38**		**>0.38**	
Crude	63	Ref	-1.8 (-18.34, 14.65)	0.823	-4.9 (-21.73, 12.00)	0.565	9.3 (-7.97, 26.63)	0.284
Model A^1^	57	Ref	-3.2 (-21.01, 14.61)	0.719	-6.8 (-25.19, 11.50)	0.456	9.6 (-9.30, 28.56)	0.311
Model B^2^	52	Ref	-18.2 (-37.96, 1.62)	0.070	-12.3 (-34.98, 10.37)	0.257	-4.2 (-26.88, 18.51)	0.708
**MEHHP (ng/mL)**		**<0.18**	**0.18 – 0.27**		**0.28 – 0.39**		**>0.39**	
Crude	63	Ref	-4.0 (-21.73, 13.73)	0.653	-3.1 (-22.87, 16.77)	0.758	2.7 (-16.20, 21.54)	0.777
Model A^1^	57	Ref	-1.9 (-21.06, 17.23)	0.841	-1.7 (-22.42, 19.11)	0.873	7.8 (-12.74, 28.36)	0.448
Model B^2^	52	Ref	-2.4 (-26.23, 21.51)	0.841	1.3 (-24.08, 26.60)	0.919	4.6 (-22.28, 31.50)	0.728
**MEOHP (ng/mL)**		**<0.17**	**0.17 – 0.23**		**0.24 – 0.41**		**>0.41**	
Crude	63	Ref	15.2 (-2.37, 32.71)	0.089	8.8 (-9.37, 26.97)	0.336	12.2 (-5.97, 30.37)	0.184
Model A^1^	57	Ref	13.6 (-4.66, 31.95)	0.140	7.9 (-10.50, 26.30	0.392	15.9 (-2.98, 34.75)	0.097
Model B^2^	52	Ref	3.8 (-19.48, 27.16)	0.738	-6.1 (-31.51, 19.31)	0.626	-1.8 (-26.20, 22.58)	0.880
**PFOS (ng/L)**		**<1026**	**1026 – 1600**		**1601 – 2175**		**>2175**	
Crude	62	Ref	-22.1^*^ (-38.75, -5.48)	0.010	-16.8^*^ (-33.47, -0.21)	0.047	-11.8 (-29.14, 5.64)	0.181
Model A^1^	57	Ref	-22.8^*^ (-39.83, -5.83)	0.010	-18.0 (-36.84, 0.79)	0.060	-13.9 (-31.56, 3.81)	0.121
Model B^2^	52	Ref	-7.9 (-31.56, 15.74)	0.499	-16.5 (-40.32, 7.34)	0.167	-9.6 (-32.57, 13.31)	0.396
**PFOA (ng/L)**		**<591**	**591 – 865**		**866 – 1200**		**>1200**	
Crude	62	Ref	-20.3^*^ (-37.29, -3.24)	0.021	-12.2 (-27.15, 2.80)	0.109	-11.3 (-28.29, 5.80)	0.190
Model A^1^	57	Ref	-16.8 (-35.09, 1.54)	0.072	-7.7 (-23.93, 8.46)	0.341	-9.2 (-27.09, 8.79)	0.310
Model B^2^	52	Ref	7.9 (-18.04, 33.92)	0.536	-2.1 (-20.94, 16.78)	0.823	6.2 (-16.08, 28.50)	0.572

^1^Adjusted for thyroid gland problems, use of thyroid medication, and birth weight. Caesarean section was constant and not included in the model.

^2^Model A + additionally adjusted for gestational weight gain, gestational age, parity, smoking, alcohol, maternal BMI, and maternal age at birth.

^*^p < 0.050.

**Table 4:** **Regression coefficients for various exposures in girls (in quartiles) and T4 (nmol/L)**

	n	Q1	Q2		Q3		Q4	
Compound			β (95% CI)	p-value	β (95% CI)	p-value	β (95% CI)	p-value
**DDE (Total, ng/L)**		**<41.80**	**41.80 – 74.50**		**74.51 – 107.50**		**>107.50**	
Crude	70	Ref	0.7 (-16.17, 17.48)	0.938	3.9 (-14.66, 22.47)	0.675	20.2^*^ (1.59, 38.71)	0.034
Model A^1^	65	Ref	3.0 (-14.66, 20.69)	0.734	2.3 (-17.14, 21.80)	0.811	24.4^*^ (2.30, 46.47)	0.031
Model B^2^	61	Ref	8.7 (-10.00, 27.45)	0.350	8.6 (-13.00, 30.18)	0.424	24.8^*^ (0.79, 48.75)	0.043
**MECPP (ng/mL)**		**<0.22**	**0.22 – 0.27**		**0.28 – 0.38**		**>0.38**	
Crude	63	Ref	-1.6 (-22.55, 19.30)	0.877	-12.0 (-36.62, 12.62)	0.333	11.3 (-13.37, 35.87)	0.363
Model A^1^	57	Ref	3.4 (-18.62, 25.37)	0.759	-10.3 (-38.03, 17.52)	0.461	16.3 (-11.69, 44.36)	0.247
Model B^2^	52	Ref	6.2 (-24.71, 37.04)	0.685	-14.5 (-45.49, 16.44)	0.344	14.0 (-14.01, 42.07)	0.314
**MEHHP (ng/mL)**		**<0.18**	**0.18 – 0.27**		**0.28 – 0.39**		**>0.39**	
Crude	63	Ref	-9.3 (-34.00, 15.47)	0.456	-8.0 (-28.68, 12.68)	0.441	-3.2 (-30.24, 23.91)	0.815
Model A^1^	57	Ref	-7.8 (-35.52, 20.00)	0.575	-8.1 (-29.95, 13.66)	0.456	-1.5 (-29.50, 26.41)	0.912
Model B^2^	52	Ref	-5.7 (-49.41, 38.11)	0.793	-11.2 (-35.27, 12.94)	0.350	-0.4 (-30.20, 29.43)	0.979
**MEOHP (ng/mL)**		**<0.17**	**0.17 – 0.23**		**0.24 – 0.41**		**>0.41**	
Crude	63	Ref	-10.5 (-32.11, 11.08)	0.333	6.6 (-18.83, 32.07)	0.604	-10.2 (-30.73, 10.30)	0.322
Model A^1^	57	Ref	-12.8 (-36.53, 10.91)	0.283	2.5 (-30.01, 34.94)	0.879	-9.7 (-32.80, 13.37)	0.401
Model B^2^	52	Ref	-19.6 (-47.55, 8.30)	0.161	1.6 (-35.19, 38.30)	0.932	-12.5 (-40.01, 19.92)	0.357
**PFOS (ng/L)**		**<1026**	**1026 – 1600**		**1601 – 2175**		**>2175**	
Crude	62	Ref	-3.3 (-24.28, 17.65)	0.752	-2.7 (-27.42, 22.00)	0.826	9.5 (-10.46, 29.37)	0.345
Model A^1^	57	Ref	2.3 (-21.13, 25.69)	0.845	-1.4 (-27.00, 24.17)	0.912	10.2 (-10.90, 31.24)	0.336
Model B^2^	52	Ref	-1.3 (-30.45, 27.94)	0.930	4.5 (-25.95, 34.92)	0.766	15.9 (-10.67, 42.40)	0.231
**PFOA (ng/L)**		**<591**	**591 – 865**		**866 – 1200**		**>1200**	
Crude	62	Ref	-3.6 (-23.09, 15.90)	0.713	2.6 (-19.40, 24.54)	0.815	22.8^*^ (0.86, 44.79)	0.042
Model A^1^	57	Ref	-1.5 (-22.39, 19.40)	0.886	11.3 (-14.23, 36.79)	0.378	27.8^*^ (5.03, 50.64)	0.018
Model B^2^	52	Ref	-5.9 (-26.75, 14.94)	0.566	11.8 (-19.08, 42.72)	0.439	38.6^*^ (13.34, 63.83)	0.004

^1^Adjusted for thyroid gland problems, use of thyroid medication, and birth weight. Caesarean section was constant and not included in the model.

^2^Model A + additionally adjusted for gestational weight gain, gestational age, parity, smoking, alcohol, maternal BMI, and maternal age at birth.

^*^p < 0.05

**Table 5:** **Regression coefficients for PCB-153 (<LOQ vs. >LOQ) and T4 (nmol/L), stratified for gender**

	N	<LOQ	>LOQ	
			β (95% CI)	P-value
**Male**				
Crude	48	Ref.	3.3 (-9.58, 16.20)	0.607
Model A^1^	45	Ref.	4.2 (-9.63, 18.05)	0.542
Model B^2^	42	Ref.	-0.6 (-19.02, 17.79)	0.945
**Female**				
Crude	48	Ref.	-4.5 (-22.54, 13.55)	0.618
Model A^1^	45	Ref.	-7.4 (-28.59, 13.78)	0.483
Model B^2^	42	Ref.	-17.2 (-43.52, 9.04)	0.188

^1^Adjusted for thyroid gland problems, use of thyroid medication, and birth weight. Caesarean section was constant and not included in the model.

^2^Model A + additionally adjusted for gestational weight gain, gestational age, parity, smoking, alcohol, maternal BMI, and maternal age at birth.

## Discussion

The objective of the current study was to investigate the association between prenatal exposure to various endocrine disrupting chemicals measured in cord blood and breast milk, and T4 levels determined in heel prick blood spots. Girls in the highest quartile of DDE and PFOA exposure showed increased T4 levels compared to the lowest quartile, a difference which remained significant after fully adjusting the model for covariates. For PFOA, the highest exposed group of girls had a mean T4 level which was 38.6 nmol/L higher than the reference group, a difference which is quite large considering that the range in T4 values in this cohort was 96 nmol/L. In boys a lower T4 level was seen in the second quartile of exposure for both PFOS and PFOA, however after adjusting the models, these associations attenuated and were no longer significant. For the DEHP metabolites as well as for PCB no association with T4 was observed in either boys or girls.

The positive association between DDE exposure and T4 in the highest exposed quartile of girls is not in line with what has been reported in other observational studies [16, 28, 29], which either report no association or an inverse association. It should be noted that Maervoet et al. and Asawasinsopon et al. determined fT4 or T4 in cord blood, which may have confounded their results as cord blood is sampled directly after birth and T4 levels may have been elevated due to stress of the delivery (median total T4 level in the study of Asawasinsopon et al. was 111.3 nmol/L). Furthermore DDE levels were higher in both these cohorts (189 ng/g lipid and 742 ng/g lipid for Maervoet et al. and Asawasinsopon respectively). Also in animal studies disruption of TH by DDE is reported, although generally also an inverse association is observed [30, 31]. However, due to the relatively small study population with less than 40% girls, results of the current study should also be interpreted with caution. As limited data on the effect of intra-uterine exposure to EDCs on thyroid function in newborns are available, and as results thus far are equivocal, more research is warranted to clarify dose–response associations between prenatal DDE exposure and T4 in newborns.

Levels of PFOS and PFOA observed in this study population were relatively low compared to what has been reported for other birth cohorts [15, 32–34]. With the exception of Kim et al. [15], none of these studies determined associations between exposure and thyroid hormone levels. Kim et al. observed no associations between cord blood PFOS or PFOA concentrations and T4 or TSH in the newborn. Concentrations of exposures were similar to the current study, however analyses were not stratified for gender, which may explain discrepancies with our results. We observed that girls with a relatively high PFOA exposure had a T4 level which was significantly higher than their peers with low exposure. These gender dimorphic associations have also been seen by Knox et al. [35], who determined PFOS and PFOA in serum of adults and found both compounds to be associated with increases in serum T4 [35]. They furthermore observed gender specific interactions for PFOS, but not PFOA, and T4, with women having higher T4 levels than men across all quartiles of exposure.

The differences in T4 levels for PFOA exposure in girls are relevant as they may potentially have implications for their health later in life. In a Spanish cohort, low free T4 and high TSH levels within the normal range were associated with symptoms of attention deficit hyperactivity disorder (ADHD) in four year olds [36]. A similar observation was made in subjects with resistance to thyroid hormone, in whom total T4 concentrations correlated positively with symptoms of hyperactivity [37]. Though the study by Alvarez-Pedrerol et al. was a cross-sectional study, the association between symptoms of ADHD and thyroid hormones in the normal range may be an indication of the sensitivity of the process of neurodevelopment at young age. None of the subjects in the current study had deviating or dubious T4 levels according to the cut-offs used in the Dutch neonatal screening program [38]. It would be interesting to follow these children up at later age to see if there is a relation between early life exposure and prevalence of neurodevelopmental disorders, and if so, if these associations are mediated by thyroid hormones.

It has been shown in experimental studies that PFAAs may displace T4 from binding proteins such as albumin [39–41] or transthyretin [42], which would result in an increase in fT4 and a subsequent decrease in T4, opposite from our observations. This has been however predominantly shown in vitro. In vivo studies are few, and it has furthermore been suggested that animals react differently to endocrine disrupting chemicals, either having a higher tolerance [43], as well as having a TH status more sensitive to exogenous compounds [44]. It would therefore be very interesting to measure both T4 and thyroid binding proteins in humans, however this information was not available in the current study. Further research is required to elucidate how PFAAs interact with thyroid hormones.

Data within this cohort was collected prospectively, and though small, this study population was very homogenous as all women were of Dutch origin and the majority was high educated (64% having a bachelor or master degree). Associations are therefore less likely to be confounded by demographic or socio-economic factors. Covariates were carefully selected based on literature, however the association between fetal T4 and gestational weight gain has not been reported on frequently, which might make inclusion of this variable in the models questionable. However in a recently published study by Pop et al. [25], fetal fT4 concentrations were associated with high weight gain during pregnancy [45]. Furthermore Verner et al. [46] reported that gestational weight gain is associated with lipophilic compounds in particular, implying that it should be considered as a potential confounder. Also birth weight was included as a covariate, however associations between birth weight and thyroid hormones have mainly been reported for maternal as opposed to offspring thyroid hormones. We therefore performed sensitivity analysis by excluding both variables independently from the models, which did not affect results. We furthermore did not adjust our analysis for time of heel prick sampling, which was done between 4–7 days after birth. T4 levels peak at 24 hours after birth and decrease slowly over the following days [47]. Even though changes in T4 levels are small during the period heel prick samples are collected, this may have obscured effects or may have resulted in non-relevant findings.

The sample size in this study was small, and power was reduced by dividing subjects in quartiles based on exposure, which was necessary as there was no linear association between outcome and exposure. Moreover results were stratified for gender, and models were adjusted for several confounders, which also reduced power of the tests. Pooling the children into one group and not stratifying for gender was considered, however as these compounds are known to disrupt the endocrine system, which is gender-specific in itself, it was decided to perform stratified analysis. Though there are no indications we know of for gender-specific effects regarding disruption of thyroid hormones, our results only showed effects for girls. As the standard errors of the models were not affected after stratification, it was decided to present results separately for boys and girls. We have not used corrections for multiple testing, such as a Bonferroni correction, because this would further reduce the power of our statistical tests, but we are aware that some of the findings may be false positive findings.

The small sample size was partly due to 51 participants for whom no samples or insufficient volumes of cord blood were available. Most of the participants gave birth at home, and collection of cord blood was not always possible due to emergency transport to the hospital where the participants were referred to the obstetrician, or errors occurred in transport of samples to the laboratory. For some participants no breast milk was available, as not all children received breast feeding.

DDE levels determined in milk samples, approximately one third of samples, were converted to cord blood values by means of a factor derived from what has been previously published in a meta-analysis of twelve birth cohorts. Though there is no standard conversion factor, we considered this the best option as it was based on cohorts from Europe. Nonetheless, it may have attenuated associations.

No associations between PCB-153 and T4 were observed in both boys and girls. This was in line with a similar study in a Canadian birth cohort, despite their relatively high exposure to this compound [48]. On the other hand, an inverse association between fT4 and PCB-153 was reported for a Belgian cohort, in which exposure levels were comparable to levels in our study population [28]. It must be noted that for both the Belgian and the Canadian cohort, thyroid hormones were determined in cord blood, and comparison of results for PCB-153 is therefore complicated. Also for each of the DEHP metabolites no effects on T4 were observed. Exposure was determined in cord blood and breast milk, which is relatively novel as in most studies urine is used. We were able to quantify levels in the majority of the samples, as the LOQ was very sensitive and in some samples as low as 0.13 ng/mL, compared to a limit of detection of 0.2 ng/mL which was for example reported by Olsén et al. who determined DEHP metabolites in serum of elderly people in Sweden [49]. Though there are several studies in adults, only one study is available which related serum T4 levels to phthalate levels in urine of four to nine year old children [50]. No effect of exposure on T4 was reported, however, various phthalates showed a positive association with levels of free and total T3, indicating that phthalates may potentially also interact with TH. Experimental evidence for this is however insufficient. As application of phthalates is widespread, future studies should aim to include these compounds when investigating the effect of environmental exposures on thyroid function.

## Conclusions

This study showed that DDE and PFAAs may be associated with T4 in a sex-specific manner. The study population was relatively small, therefore results should be interpreted with caution, and confirmation from larger studies is warranted. Thyroid hormones are very important during developmental stages in life, and even subtle changes may have an impact on child health. More research is warranted, as studies on the role of environmental contaminants in this area are still limited.

## Electronic supplementary material

Additional file 1: **Prenatal exposure to endocrine disrupting chemicals in relation to thyroid hormone levels in infants.** (DOCX 25 KB)

## Abbreviations

BMI: Body mass index
DDE: Dichlorodiphenyldichloroethylene
DEHP: Di-2-ethylhexyl phthalate
EDCs: Endocrine disrupting chemicals
fT4: Free thyroxine
HCB: Hexachlorobenzene
LOQ: Limit of quantification
MECPP: Mono(2-ethyl-5-carboxypentyl)phthalate
MEHHP: Mono(2-ethyl-5-hydroxyhexyl)phthalate
MEHP: Mono(2-ethylhexyl)phthalate
MEOHP: Mono(2-ethyl-5-oxohexyl)phthalate
PBDE: Polybrominated diphenylether
PCB-153: Polychlorinated biphenyl-153
PFAAs: Perfluorinated alkyl acids
PFOA: Perfluorooctanoic acid
PFOS: Perfluorooctanesulfonic acid
T3: 3,5,3′-triiodothyronine
T4: Thyroxine
TH: Thyroid hormones
TSH: Thyroid stimulating hormone.
