# Supplementary material for: Prenatal exposure to endocrine disrupting chemicals in relation to thyroid hormone levels in infants – a Dutch prospective cohort study
Source: Environ Health. 2014 Dec 10;13:106. doi: 10.1186/1476-069X-13-106 (PMC4293007; doi:10.1186/1476-069X-13-106)
Supplement: Supplementary file 1 — Additional file 1: Prenatal exposure to endocrine disrupting chemicals in relation to thyroid hormone levels in infants. (DOCX 25 KB) [file 12940_2014_816_MOESM1_ESM.docx]

# Supplemental Material - Prenatal exposure to endocrine disrupting chemicals in relation to thyroid hormone levels in infants

Marijke de Cock, MSc^1^, Michiel R. de Boer, PhD^2^, Marja Lamoree, PhD^3^, Juliette Legler, PhD^3^, Margot van de Bor, MD, PhD ^1^

**Contents**

[Analysis of organochlorine pesticides and PCB 153 2](#_Toc391621823)

[Analysis of DEHP metabolites 2](#_Toc391621824)

[Analysis of PFOS and PFOA 3](#_Toc391621825)

[Determination of lipid content of breast milk and cord plasma samples 3](#_Toc391621826)

[QA/QC procedures 4](#_Toc391621827)

[References 5](#_Toc391621828)

## Analysis of organochlorine pesticides and PCB 153

For the determination of the organochlorine pesticides (HCB and p,p’-DDE) and PCBs (PCB153), 3 ml of cord plasma or 12 ml breast milk were dried with Kieselguhr after addition of the internal standard (^13^C_6_-HCB, ^13^C_10_-PCB153 (from Cambridge Isotope Laboratories) and BDE58). The dried samples were extracted with 30 ml hexane/dichloromethane (7:3 v/v) for 30 minutes by sonication. The extracts were purified over sulphuric acid silica columns (5 g, 40% sulphuric acid w/w) that were eluted with 30 ml hexane/dichloromethane (7:3 v/v). The eluates were evaporated under a gentle stream of nitrogen. Finally, the extracts were quantitatively transferred to GC-vials, with a final volume of 100 µl iso-octane.

The samples were analyzed using an Agilent 6890 GC with a 5975 Mass Spectrometric Detector in negative chemical ionization mode. The GC was equipped with a CPSil-8 CB column (Varian, 50 m x 0.25 mm ID x 0.25 µm film thickness). The samples were injected in pulsed splitless mode with the injector at 275°C. The oven was programmed as follows: initial temperature: 90°C for 3 min; then to 210°C at 30°C/min and held for 20 min; finally to 290 °C at 5°C/min and held for 3 minutes (total run time 45 minutes). Carrier gas was helium at a flow rate of 2.7 ml/min. The compounds of interest were quantified by using m/z 283.8 for HCB; m/z 359.8 for PCB 153 and m/z 35 for p,p’-DDE (compound confirmation by GC-MS/EI measurement of m/z 246).

### Analysis of DEHP metabolites

To assess the exposure to DEHP, the secondary metabolites MEOHP (mono(2-ethyl-5-oxohexyl) phthalate), MEHHP (mono (2-ethyl-5-hydroxyhexyl) phthalate) and MECPP (mono(2-ethyl-5-carboxypentyl) phthalate) were quantitatively determined. The remaining enzymatic activity in the sample material was quenched by adding 0.02 ml 1 M phosphorous acid to 0.3 ml cord plasma or 0.04 ml 1 M phosphorous acid to 0.5 ml breast milk. The mixtures were then sonicated for 5 minutes. To adjust the pH of the plasma and milk samples to 6.2, 0.04 ml and 0.06 ml 1M NaOH was added to the samples, respectively. After the addition of the internal standard (^13^C_4_ – MEOHP, ^13^C_4_ – MEHHP, ^13^C_4_ – MECPP and MEHP- d_4_, all from Cambridge Isotope Laboratories), 5 μl β–glucuronidase from E. Coli K12 (from Roche) in 0.2 ml 2.5 M ammonium acetate buffer (pH 6.2) was added. The samples were incubated for 90 minutes at 37°C. The completeness of the deconjugation step was checked by adding 4-methylumbelliferone-glucuronide to each sample. The β–glucuronidase activity was stopped by adding 0.06 ml formic acid to the plasma samples and 0.06 ml ammonium hydroxide to the milk samples followed by sonication for 15 minutes and overnight storage at -20°C.

The deconjugated plasma samples were thawed and centrifuged for 15 minutes at 17000 rpm. The supernatants are transferred to a vial to which 0.2 ml water is added. The milk samples were also centrifuged for 15 minutes (17000 rpm) to remove the lipids. The remaining part was extracted by solid phase extraction (SPE). The Oasis MAX 3cc 60 mg cartridges were conditioned with 3 ml methanol and 3 ml milliQ. After the samples were loaded at 1 ml/min, the cartridges were washed with 1 ml 5% ammonium hydroxide and 1 ml 75% tetrahydrofuran in methanol. The metabolites were eluted from the cartridges with 5 ml 5% formic acid in methanol. To the eluates 0.2ml milliQ was added before evaporation to a volume of 0.2 ml. Finally, 0.2 ml 4% ammonium hydroxide and 0.2ml milliQ were added to the extracts.
The obtained extracts were injected onto a RAM (restricted access material) phase cartridge (LiChrospher RP-8 ADS, 25 μm, 25 x 4 mm). After trapping and cleanup, the analytes were eluted in backflush mode and transferred to the analytical column (Luna Phenyl-hexyl 75 x 4.6 mm) using a gradient of 0.1 % acetic acid and acetonitrile with a flow rate of 0.25 ml/min. The LC system was an Agilent 1200 Series (Palo Alto, CA, USA) coupled with an Agilent 6410 electro spray interface (ESI) operated in the negative ion mode prior to triple-quadrupole mass spectrometric detection. For MECPP, the ion transition used for quantification was m/z 307.1 – m/z 159.1, for MEHHP the ion transition m/z 293.1 – m/z 145.1, for MEOHP the ion transition m/z 291.1 – m/z 143.1, and for MEHP the ion transition m/z 277.1 – m/z 134.1 were used.

### Analysis of PFOS and PFOA

For the determination of perfluorooctane sulfonate (PFOS) and perfluorooctanoic acid (PFOA), the breast milk samples were thawed and homogenized after stabilizing them at a temperature of 38°C. Subsequently, aliquots of 0.5ml of each sample were taken for analysis. The sample was sonicated for 30 min after addition of the internal standards (^13^C_4_-PFOA and ^13^C_4_PFOS, from Wellington Laboratories) and 0.5ml 1M formic acid. Solid phase extraction was carried out using 1cc, 30 mg Oasis Wax cartridges. The cartridges were conditioned with 1 ml methanol and 1 ml MilliQ. The samples were loaded at a flow rate of 1ml/min along with the rinse volume of the sample tube, i.e. 1 ml 25 mM ammonium acetate pH4. The cartridges were washed with 1ml 25 mM ammonium acetate pH4 and 0.5 ml 25% tetrahydrofuran in methanol. The PFASs were eluted from the cartridge with 0.4 ml 1% NH_4_OH in methanol and 0.4 ml 0.1M formic acid was added to the eluate.

For the analysis of the cord plasma samples 0.2 ml cord plasma was mixed with 0.2 ml methanol. After addition of the internal standard the mixture was homogenized and centrifuged for 15 minutes at 17000 rpm. The obtained supernatants were diluted and mixed with 0.5 ml 0.1 M formic acid.

The total volume of the extracts was injected and the PFASs are trapped on a C8-column (Xterra MS C_8_, 10 mm x 4.6 mm, particle size 5 µm) in an on-line system with the analytical column (Betasil C8, 50 mm x 2.1 mm, particle size 3 µm). Subsequently the PFASs are eluted from the trapping column and separated on the analytical column using gradient elution at a flow rate of 0.3 ml/min. For the gradient 20 mM NH_4_AC pH4 and acetonitrile were used. The LC system was an Agilent 1200 Series (Palo Alto, CA, USA) coupled with an Agilent 6410 electro spray interface (ESI) operated in the negative ion mode prior to triple-quadrupole mass spectrometric detection. For PFOA, the ion transition used for quantification was m/z 413 – m/z 369, and for PFOS the ion transition m/z 499 – m/z 80 was used.

### Determination of lipid content of breast milk and cord plasma samples

In the breast milk samples, the lipid content was determined using a method adapted from Manirakiza et al. ([1](#_ENREF_1)). To 6 ml of sample, 13 ml of isoproanol and 15 ml of cyclohexane were added. The mixture was shaken vigorously for 5 minutes. Subsequently, 10 ml water was added. The cyclohexane phase containing the lipids was separated from the mixture. This procedure was repeated with a mixture of 15 ml isopropanol and cyclohexane (13:87 v/v). After combining the two cyclohexane fractions, the solvent was evaporated by a gentle nitrogen stream till dryness. The remaining lipids were gravimetrically determined after drying for 1 hr at 105°C.

In cord plasma, the lipid content was determined using standard protocols by the measurement of the triglycerides and cholesterol at the clinical laboratory of the academic hospital (ISO 15189 accredited) of the VU University, VUmc (Amsterdam, NL).

### QA/QC procedures

For all the analyses described, no Certified Reference Materials (CRMs) were available. Therefore, in every measurement series (< 16 samples) a procedure blank, an enriched sample (similar/same matrix) and a sample from a previous series were included. The analytical values obtained for the enriched sample should fall within 20% of the known level. The re-analysis of a sample from a previous series, should give a result with z-values < |2|.

In case the procedure blank revealed that the compound(s) to be analyzed were present above the limit of detection (LOD), the series was repeated. All results were corrected using the long term average blank value. The obtained blank data were used for the determination of the LOD (as 3* standard deviation in the blank) and the limit of quantitation (LOQ, defined as 3*LOD).

The performance characteristics of all methods – LOD, recovery and repeatability for breast milk and cord plasma - are given in table S6.1.

Table S6.1. Performance characteristics of all the methods used for the assessment of exposure markers in breast milk and cord plasma

|  | Milk | | | Plasma | | |
| --- | --- | --- | --- | --- | --- | --- |
|  | LOD | Recovery | Repeatability | LOD | Recovery | Repeatability |
|  | (pg/ml) | (%) | (%) | (pg/ml) | (%) | (%) |
| PCB153 | 10 | 96 (89-105) | 7 | 13 | 105 (97-113) | 5 |
| 4,4’-DDE | 17 | 98 (91-109) | 9 | 13 | 96 (82-119) | 13 |
| PFOS | 1.4 | 105 (89–105) | 9 | 5 | 86 (81-90) | 3 |
| PFOA | 0.4 | 102 (98–104) | 8 | 7 | 84 (75-90) | 6 |
| MECPP | 20 | 94 (84-104) | 5 | 40 | 100 (96-106) | 3 |
| MEHHP | 30 | 95 (78-107) | 11 | 10 | 92 (81-89) | 4 |
| MEOHP | 30 | 91 (67-113) | 16 | 20 | 92 (82-98) | 6 |
| MEHP | 60 | 96 (65-120) | 16 | 30 | 75 (70-83) | 5 |

# References

**1.** Manirakiza P, Covaci A, Schepens P 2001 Comparative Study on Total Lipid Determination using Soxhlet, Roese-Gottlieb, Bligh &amp; Dyer, and Modified Bligh &amp; Dyer Extraction Methods. Journal of Food Composition and Analysis **14**:93-100.
